# Medical Mistrust and Stigma Associated with COVID-19 Among People Living with HIV in South Africa

**DOI:** 10.1007/s10461-021-03307-8

**Published:** 2021-05-17

**Authors:** Jana Jarolimova, Joyce Yan, Sabina Govere, Nompumelelo Ngobese, Zinhle M. Shazi, Anele R. Khumalo, Bridget A. Bunda, Nafisa J. Wara, Danielle Zionts, Hilary Thulare, Robert A. Parker, Laura M. Bogart, Ingrid V. Bassett

**Affiliations:** 1 Division of Infectious Diseases, Massachusetts General Hospital, Boston, USA; 2 Medical Practice Evaluation Center, Massachusetts General Hospital, Boston, USA; 3 Biostatistics Center, Massachusetts General Hospital, Boston, USA; 4 AIDS Healthcare Foundation, Durban, South Africa; 5 Center for AIDS Research (CFAR), Harvard University, Boston, USA; 6 Harvard Medical School, Boston, USA; 7 RAND Corporation, Santa Monica, USA

**Keywords:** COVID-19, Medical mistrust, Stigma, HIV, South Africa

## Abstract

We evaluated COVID-19 stigma and medical mistrust among people living with HIV in South Africa. We conducted telephone interviews with participants in a prospective study of a decentralized antiretroviral therapy program. Scales assessing medical mistrust, conspiracy beliefs, anticipated and internalized stigma, and stereotypes specific to COVID-19 were adapted primarily from the HIV literature, with higher scores indicating more stigma or mistrust. Among 303 participants, the median stigma summary score was 4 [interquartile range (IQR) 0–8; possible range 0–24] and 6 (IQR 2–9) for mistrust (possible range 0–28). A substantial proportion of participants agreed or strongly agreed with at least one item assessing stigma (54%) or mistrust (43%). Higher COVID-19 stigma was associated with female gender and antecedent HIV stigma, and lower stigma with reporting television as a source of information on COVID-19. Further efforts should focus on effects of stigma and mistrust on protective health behaviors and vaccine hesitancy.

## Introduction

Over 100 million people worldwide have been infected with Severe Acute Respiratory Syndrome-Coronavirus 2 (SARS-CoV-2) since December 2019, leading to over two million deaths from COVID-19 [1]. South Africa has been significantly affected, with nearly 1.5 million COVID-19 cases by early February 2021 [1]. Non-pharmaceutical interventions, such as social distancing, wearing masks, and travel restrictions, are effective at reducing community spread of COVID-19 [2], and along with wide-spread testing and vaccine development, have formed the cornerstone of COVID-19 pandemic control worldwide. While non-pharmaceutical interventions, testing, and vaccination hold promise to curb the pandemic, these strategies rely in large part on individual uptake and adherence. Understanding the drivers of individual- and community-level adoption of preventive measures is critical to ongoing efforts to control the spread of COVID-19. Stigma and medical mistrust may impact COVID-19 protective health behaviors, particularly among populations already experiencing stigma, such as people living with HIV (PLWH).

Stigma—a social process that leads to devaluation or discrediting based on particular attributes [3, 4]—has been identified as a barrier to protective health behaviors across a range of health conditions [5–10]. Health-related stigma has been well characterized along the continuum of health-seeking behaviors for HIV, with impacts of HIV stigma ranging from avoidance of HIV testing [6] to decreased rates of antiretroviral therapy initiation and virologic suppression [11]. Since early in the COVID-19 pandemic, stigmatizing beliefs and behaviors towards people of Asian descent, health workers, COVID-19 patients, and marginalized populations have been reported, and have even culminated in violent action [12–18]. Stereotypes (beliefs about characteristics of a stigmatized group and its members [19]) and anticipated stigma (the expectation of future discrimination [19]) related to COVID-19 have been found to impact willingness to undergo COVID-19 testing [20]. Stigma is increasingly understood as intersectional [21], influenced by drivers such as misinformation, historical trauma, or systemic racism that can fuel stigma related to multiple health conditions, with the intersection of multiple stigmas in turn leading to social, economic, and health-related outcomes [22]. Understanding COVID-19 stigma among PLWH, a population already experiencing health-related stigma, is thus important for understanding the potential impacts of stigma on health-seeking behavior.

Medical mistrust may also undermine efforts to curb the pandemic by influencing health-protective behaviors and exacerbating existing inequities. Medical mistrust is defined as active distrust in healthcare systems and medical providers with the belief that they are acting against one’s best interest [23–25]. By contrast, trust in the healthcare system is not simply the opposite of mistrust but implies the belief that organizations or providers are acting in one’s best interest [24, 26]. Similar to stigma, both medical mistrust and conspiracy beliefs have been associated with decreased health-seeking behavior and poor health outcomes across a wide range of health conditions [27–33], and studies have demonstrated the mediating role of mistrust in the association between stigma or discrimination and health behaviors [34, 35]. Medical mistrust, and the related concept of conspiracy beliefs—theories about significant events that implicate dominant and usually malicious groups or individuals [36]—are rooted in historical mistreatment by medical institutions, systemic racism, and the perception and experience of discrimination in healthcare [25, 26, 37]. Medical mistrust and conspiracy beliefs can additionally stem from misinformation [25]. In South Africa, medical mistrust and conspiracy theories have been fueled by a long-standing history of racist policies and years of targeted government misinformation regarding the HIV epidemic [38, 39]. Since the beginning of the COVID-19 pandemic, there has been a preponderance of misinformation worldwide, often spread by social media [40–44], contributing to conspiracy theories [45, 46] and creating risk for increased medical mistrust.

Impacts of stigma and medical mistrust on preventive behaviors specific to COVID-19 have been reported, including protective health behaviors (e.g. social distancing, hand washing, wearing a mask) [36, 45] and vaccine hesitancy [47]. For PLWH, stigma and medical mistrust related to COVID-19 may exacerbate pre-existing impacts of HIV stigma and medical mistrust on health protective behaviors. Uncertainty about the risk and severity of COVID-19 and HIV coinfection could additionally fuel mistrust among PLWH. In South Africa, which has the world’s largest population of people living with HIV at 7.5 million people [48], the impacts of COVID-19 stigma and medical mistrust on health behaviors among PLWH could be particularly significant in national efforts to curb the spread of the pandemic. To implement effective public health messaging and interventions to promote health-protective behaviors, levels and characteristics of stigma and medical mistrust related to COVID-19 among PLWH must be better understood. We aimed to evaluate COVID-19 stigma and medical mistrust among people living with HIV enrolled in a decentralized ART distribution program in South Africa.

## Methods

### Study Population and Data Collection

We contacted a randomly selected subsample of participants enrolled in a prospective observational cohort study evaluating the implementation of the Central Chronic Medicines Dispensing and Distribution (CCMDD) program in Umlazi, KwaZulu-Natal. The CCMDD program, established by the South African National Department of Health (DOH), allows virologically stable PLWH to collect ART and/or medication for non-communicable diseases at pre-designated community-based pick-up points of their choice, including private pharmacies, churches, and schools [49]. People accessing HIV care in 9 DOH primary health clinics offering CCMDD enrollment in the urban township of Umlazi were recruited. Eligibility criteria included HIV positive serostatus, age 18 or older, and meeting clinical criteria for CCMDD participation as defined per program guidelines (not pregnant, on ART for ≥ 1 year, and virologically suppressed). Participants completed a baseline questionnaire at enrollment into the parent study assessing demographics, HIV care history, competing needs, mental health, social support, and HIV stigma.

We randomly selected 900 of the 2097 participants currently enrolled in the parent study, anticipating a 33% response rate for a target of 300 interviews. Selected participants were distributed evenly between those enrolled in the CCMDD program for 0–6 months, 6–12 months, and > 12 months. Between 28 April and 22 May 2020, bilingual research assistants contacted participants at the phone number provided at parent study enrollment and administered a questionnaire in the participants’ preferred language (isiZulu or English) to participants providing verbal consent. The questionnaire assessed participants’ sources of information about COVID-19, their concerns regarding COVID-19 and ART pick-up, changes in daily activities they had made as a result of the pandemic or national lockdown, measures of mental health and social support, medical mistrust related to COVID-19, and stigma related to COVID-19.

### Measures

#### Sociodemographic Characteristics, HIV History, and Concerns Regarding COVID-19

We obtained basic demographic data, including age, gender, education level, employment, marital status, and years on ART from the baseline questionnaire of the parent study. We assessed participants’ sources of information on COVID-19 and primary concerns regarding the pandemic at the time of the telephone interview.

#### HIV Stigma

At enrollment into the parent study, we assessed HIV stigma using the 6 questions from the Internalized AIDS-related Stigma Scale [50], along with 6 questions from the HIV stigma scale by Berger et al. [51] that highlight disclosure concerns. Response options were 1—Agree and 0—Disagree. The frequency of “agree” responses per participant was calculated out of a possible total of 12. The scale had a Cronbach’s alpha of 0.94.

#### COVID-19 Stigma

During the telephone interview, we assessed stigma related to COVID-19 infection. We utilized 6 questions adapted from previously published stigma scales for HIV and chronic illness, with responses on a 5-point Likert scale, with scores ranging 0–4. Questions were selected to assess different domains of health-related stigma, including stereotypes (three questions), internalized stigma (two questions), and anticipated stigma (one question). Two of the questions were adapted from an HIV stigma scale designed for low- and middle-income country (LMIC) settings [52]: “a person with coronavirus must have done something wrong and deserves to be punished,” and “a person with coronavirus is cursed.” Two of the questions were adapted from the Internalized AIDS-related Stigma Scale [50]: “if I have coronavirus, it would be difficult to tell other people,” and “if I had coronavirus, I would feel ashamed”; the latter question had also been incorporated into the scale designed for use in LMIC by Genberg et al. [52]. We adapted one question from a chronic illness anticipated stigma scale [53]: “If I had coronavirus, my friends and family will be angry with me.” A final question, “if someone I knew had coronavirus and recovered, I would be afraid to visit them” was designed for this study. Scores on individual items were added to create a COVID-19 stigma summary score. Participants with missing data in individual items were categorized as having a missing stigma summary score. With regard to internal consistency, the scale had a Cronbach’s alpha of 0.67.

#### Medical Mistrust Related to COVID-19

We developed eight items assessing medical mistrust, trust in the formal healthcare system to address COVID-19, and conspiracy beliefs related to the COVID-19 pandemic. Two questions assessing mistrust of the government as an agent of healthcare and conspiracy beliefs were adapted from a published scale assessing conspiracy theories around HIV [54]: “The government cannot be trusted to tell the truth about coronavirus,” and “Information about coronavirus is being withheld from the public.” The remaining items were developed for the current study. Questions assessed trust in the government to address COVID-19 (“I trust the public health measures that the government is taking to combat coronavirus”), and trust in the media as an agent of public health messaging (i.e., “I trust the information that I am hearing on the radio and TV about coronavirus,” and “I trust the information that I am reading about coronavirus”). Additional questions assessed trust in healthcare providers and in the formal healthcare sector, by comparing trust in non-Western medicine with trust in clinic-based personnel: “I trust that the doctors and nurses in clinics know how to treat coronavirus,” “I trust traditional healers more than clinic workers to treat coronavirus,” and “I trust church leaders more than clinic workers to treat coronavirus.” Response choices were on a 5-point Likert scale, from ‘strongly disagree’ to ‘strongly agree,’ with scores for each answer option ranging 0–4. To maintain measurement of medical mistrust related to the formal healthcare sector, scores were reversed for the four questions that asked about trust in public health measures, trust in public messaging, and trust of doctors and clinic workers. Scores for individual items were added to produce a medical mistrust summary score; missing responses to individual items resulted in a missing summary score.

In terms of internal consistency, the full scale had a Cronbach’s alpha of 0.63. When the item least correlated with the remainder of the scale, “I trust that the doctors and nurses in clinics know how to treat coronavirus” was removed, the Cronbach’s alpha improved to 0.72. This question was subsequently removed from further analyses of medical mistrust.

### Statistical Analysis

We used descriptive statistics [median, interquartile range (IQR), frequency] to report the characteristics of the study population at baseline and during the COVID-19 pandemic, concerns regarding the COVID-19 pandemic, and the distributions of scores for individual COVID-19 stigma and medical mistrust items and summary scores. We used univariate and multivariable logistic regression models to assess predictors of high COVID-19 medical mistrust and high COVID-19 stigma.

For the outcome variables, we defined a high COVID-19 medical mistrust score as at or above the median score of 6, and a high stigma score as a score above the median score of 4. For predictors, we defined several combined categories of concerns regarding COVID-19. Using the responses to the question, “What are your concerns about coronavirus?”, we defined ‘economic concerns’ as any one or more of the responses, “unable to work,” “food running out,” and “money running out”; and ‘infection/illness concerns’ as any one or more of the responses, “becoming infected myself” and “family member becoming infected.” We defined ‘other’ concerns as any one or more of the remaining concerns unrelated to economic hardship or infection, which primarily included lack of transport, unavailable PPE or hand sanitizer, being afraid to move around, crime, and lack of information.

All multivariable models included age and gender. Other factors with p < 0.2 in univariate logistic regression models were also included in the specific multivariable model. All reported p-values were two-tailed, and p < 0.05 was considered statistically significant. Analyses were conducted using SAS software (version 9.4, SAS Institute, Cary, NC).

### Ethical Considerations

The study protocol was approved by the Biomedical Research Ethics Committee of the University of KwaZulu-Natal and by the Partners Healthcare Institutional Review Board (protocol 2017P001690).

## Results

### Participant Characteristics and Baseline HIV Stigma

Of the 638 enrollees contacted, 303 (47%) consented to and completed the interview. Sixty-six percent were female, 302 (99.7%) identified their ethnicity as Black, median age was 36 (IQR, 31–45), and 263 (86.8%) had completed at least some secondary school education (Table 1). Median time since initiation of ART was 3.0 years (IQR 1.0–6.0). Only 6 participants (2.0%) reported any competing needs at baseline, the majority (247, 81.8%) rated their ability to take HIV medication as “excellent,” and most (286, 94.4%) reported zero missed doses of ART in the preceding 7 days. At enrollment into the parent study, 54.6% of participants endorsed zero HIV stigma items. At the time of the telephone interview during the COVID-19 lockdown, half of participants (150, 49.5%) had been enrolled in the CCMDD program for fewer than six months, and 292 (96.4%) were continuing to receive their ART through the CCMDD program.

**Table 1:** Participant demographics and characteristics at enrollment into the parent study

Characteristic	Median, [IQR] or n, (%)
Gender	
Female	200 (66.0)
Male	103 (34.0)
Age, years	36.0 [31.0–45.0]
18–25	22 (7.3)
26–40	166 (54.8)
> 40	115 (38.0)
Ethnic group (race)	
Black	302 (99.7)
Coloured	1 (0.3)
Years since initiation of ART	3.0 [1.0–6.0]
Education level	
No school	6 (2.0)
Primary	34 (11.2)
Some high school	123 (40.6)
Matric	120 (39.6)
Tertiary	20 (6.6)
Employed	
No	164 (54.1)
Yes	139 (45.9)
Marital status	
Never married	237 (78.2)
Currently married	58 (19.1)
Divorced/separated	5 (1.7)
Widowed	3 (1.0)
Ability to take HIV medication (n = 302)	
Very poor, poor, or fair	0
Good	6 (2.0)
Very good	49 (16.2)
Excellent	247 (81.8)
Doses of ART missed in the preceding 7 days	
0 doses missed	286 (94.4)
1–3 doses missed	17 (5.6)
> 3 doses missed	0
Baseline competing needs^a^	
None	297 (98.0)
Any	6 (2.0)
Number of HIV stigma items endorsed at baseline, range 0–12 (n = 293)	
0	160 (54.6)
1–6	65 (22.2)
> 6	68 (23.2)
Time enrolled in CCMDD prior to COVID-19 interview, months	7 [5–12]
0–6 months	150 (49.5)
6–12 months	81 (26.7)
> 12 months	72 (23.8)

n = **303** unless noted otherwise

^a^Whether the participant had gone without healthcare because they needed the money for basic needs, such as food, clothing, or housing, or if they had gone without basic needs because they needed the money for healthcare, in the preceding 6 months before enrollment in CCMDD

### Sources of Information and Concerns about the Pandemic

Television and radio were the most common sources of information on COVID-19, reported by 249 (82.2%) and 219 (72.3%) participants, respectively, followed by social media (60, 19.8%) and friends/family (46, 15.2%) (Table 2). Participants’ most frequent concerns regarding the COVID-19 pandemic and national lockdown were food running out (121, 39.9%), becoming infected with COVID-19 (103, 34.0%), being unable to work/losing employment or income (102, 33.7%), and money running out (87, 28.7%) (Table 2). Only 4 (1.3%) participants spontaneously reported stigma as a concern during the pandemic when unprompted.

**Table 2:** Sources of information on COVID-19 and concerns about the COVID-19 pandemic

Sources of information on COVID-19	n (%)
TV	249 (82.2)
Radio	219 (72.3)
Social Media	60 (19.8)
Friends/family	46 (15.2)
Newspapers/news websites	38 (12.5)
Clinics (materials, staff)	32 (10.6)
DOH/Government website	30 (9.9)
At work	15 (5.0)
Other websites	8 (2.6)
Other	19 (6.3)

Concerns about the COVID-19 pandemic	
Food running out	121 (39.9)
Becoming infected myself	103 (34.0)
Unable to work	102 (33.7)
Money running out	87 (28.7)
Family member becoming infected	54 (17.8)
Lack of transport to work, clinic, or elsewhere	39 (12.9)
Afraid to move	33 (10.9)
Unavailable sanitizer/PPE	24 (7.9)
Lack of transport to CCMDD pick-up point	9 (3.0)
Crime	8 (2.6)
Lack of information	5 (1.7)
Stigma	4 (1.3)
Other	80 (26.4)

### COVID-19 Stigma

**Fig. 1:**
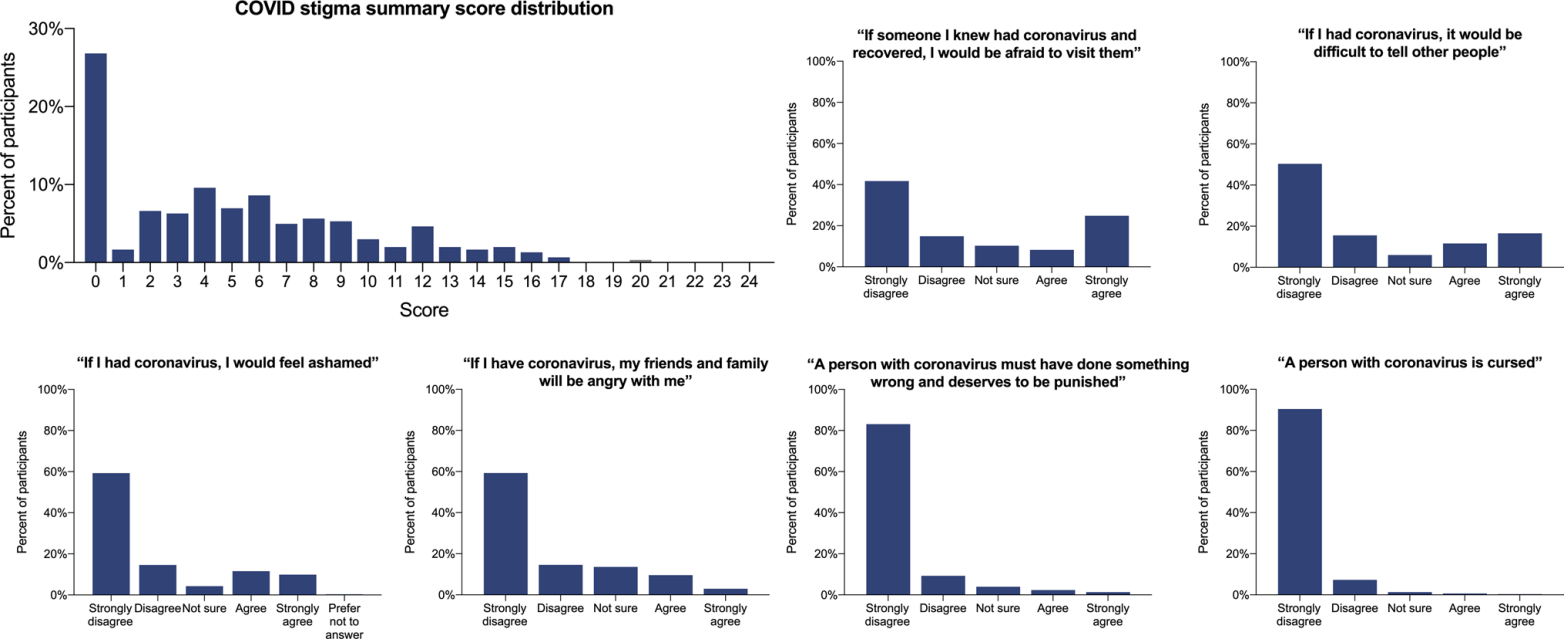
COVID-19 stigma summary scores and distributions of responses for individual stigma items

Of 301 participants with data available for all stigma items, over one quarter (81, 26.9%) of participants strongly disagreed with all items assessing stigma and thus had the lowest possible COVID-19 stigma summary score of 0. With a possible score range of 0–24, the median summary score was 4 with IQR 0–8, indicating overall low levels of stigma. The distribution of summary stigma scores and of answers for each individual stigma item are shown in Fig. 1. Of 302 participants with data available for any stigma items, a majority (164, 54.3%) agreed or strongly agreed with at least one item assessing stigma. The most frequently endorsed COVID-19 stigma item, at 33%, was, “If someone I knew had coronavirus and recovered, I would be afraid to visit them,” with the least frequently endorsed questions being, “A person with coronavirus must have done something wrong and deserves to be punished,” and “A person with coronavirus is cursed,” (4% and 1%, respectively). In multivariable analyses, female gender (aOR 1.88, 95% CI 1.10–3.20), having ‘other’ concerns about the COVID-19 pandemic (aOR 2.04, 95% CI 1.14–3.66), and endorsing greater than 6 HIV stigma items at baseline (aOR 2.09, 95% CI 1.11–3.93) were associated with having a COVID-19 stigma score above the median, while identifying television as a source of information on COVID-19 made one less likely to have a stigma score above the median (aOR 0.46, 95% CI 0.24–0.90) (Table 3).

**Table 3:** Correlates of high COVID-19 stigma, n = 291

	OR, univariate analyses	p-value	aOR, multivariable analysis	p-value
Gender (Ref = M)				
F	1.97 (1.21–3.20)	0.006	1.88 (1.10–3.20)	**0.02**
Age (Ref = > 40)				
18–25	3.62 (1.31–10.00)	0.01	2.02 (0.67–6.08)	0.21
26–40	1.54 (0.95–2.49)	0.08	1.21 (0.71–2.05)	0.49
Education level (Ref = no school)				
Primary	0.70 (0.12–3.99	0.69		
Some high school	0.81 (0.16–4.17)	0.80		
Matric	1.14 (0.22–5.89)	0.87		
Tertiary	1.57 (0.25–10.09)	0.63		
Sources of information on COVID-19				
Newspaper/news websites	0.65 (0.32–1.30)	0.22		
Radio	0.63 (0.38–1.05)	0.07	0.66 (0.37–1.15)	0.14
Television	0.50 (0.27–0.91)	0.02	0.46 (0.24–0.90)	**0.02**
DOH/government website	0.78 (0.37–1.67)	0.53		
Social media	0.89 (0.50–1.59)	0.70		
Friends/family	0.63 (0.33–1.19)	0.15	0.58 (0.28–1.19)	0.14
Clinics	0.69 (0.33–1.45)	0.33		
Other	1.77 (0.90–3.47)	0.10	1.59 (0.76–3.33)	0.22
Any economic concerns about COVID-19	0.88 (0.55–1.38)	0.57		
Infection as a concern about COVID-19	0.80 (0.50–1.26)	0.33		
Any other concerns about COVID-19	2.71 (1.58–4.66)	< 0.001	2.04 (1.14–3.66)	**0.02**
Number of HIV stigma items endorsed at baseline (Ref = 0)				
1–6	1.44 (0.81–2.57)	0.22	1.31 (0.70–2.43)	0.40
> 6	2.56 (1.42–4.61)	0.002	2.09 (1.11–3.93)	**0.02**

### COVID-19 Medical Mistrust

**Fig. 2:**
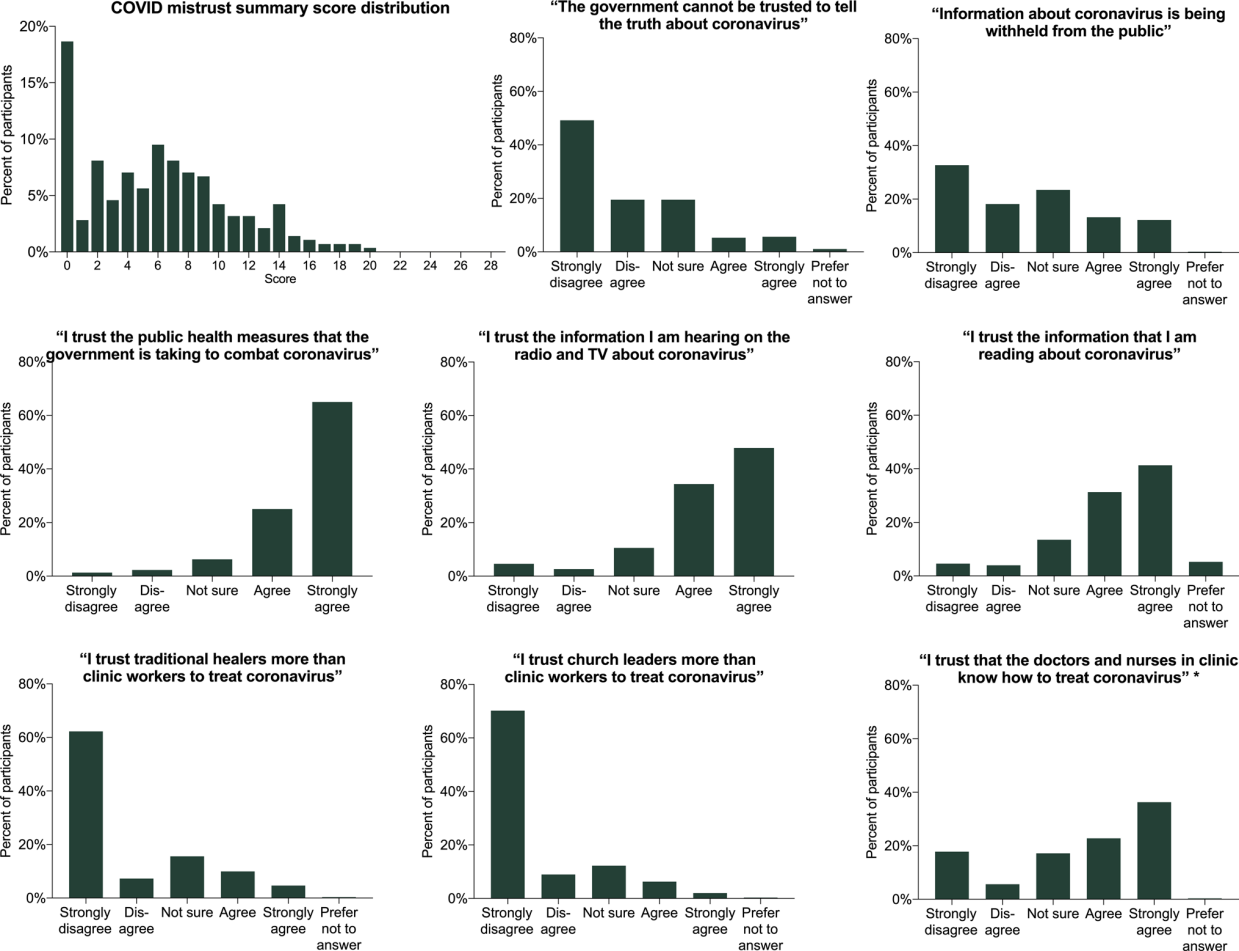
COVID-19 medical mistrust summary scores and distributions for individual mistrust items. The question, “I trust that the doctors and nurses in clinic know how to treat coronavirus” was not included in the summary score

Among 283 participants with data available for all mistrust items, the median summary score for medical mistrust related to COVID-19 was 6 (interquartile range, 2–9) on a scale from 0–28. The distribution of summary medical mistrust scores and for each answer item are shown in Fig. 2. Fifty-three participants (18.7%) had the lowest score of 0, indicating that they strongly disagreed with all mistrust items and conspiracy beliefs, and strongly agreed with all ‘trust’ items. Only 26 participants (9.2%) had a summary score above 13, and nobody had a score above 20. However, 130 participants (42.9%) among those with data on any mistrust items available agreed or strongly agreed with at least one mistrust item. In multivariable analyses, identifying illness-related concerns regarding the COVID-19 pandemic was associated with lower medical mistrust (aOR 0.53, 95% CI 0.31–0.92). Medical mistrust was otherwise not associated with gender, age, education level, sources of information on COVID-19, or other categories of concerns about the COVID-19 pandemic (data not shown).

## Discussion

This study demonstrates that early in the COVID-19 pandemic in South Africa, a significant proportion of people living with HIV enrolled in a decentralized ART delivery program endorsed aspects of COVID-19 stigma and medical mistrust. Specifically, over half of participants agreed or strongly agreed with at least one item assessing anticipated stigma, internalized stigma, or stereotypes specific to COVID-19, and over two-fifths of participants agreed or strongly agreed with at least one item assessing mistrust in the government regarding COVID-19, conspiracy beliefs, or lack of trust in public messaging or healthcare providers. However, overall levels of stigma and medical mistrust were low when summed over all measures. While this is the first study, to our knowledge, to evaluate levels of COVID-19 stigma and medical mistrust among PLWH in South Africa, we found levels of stigma similar to findings from a general United States population [20], while levels of mistrust were somewhat lower than in a study of Black Americans living with HIV [47] and among the general population in the United Kingdom [55]. In light of the known negative associations of both stigma and mistrust with health-protective behaviors and health outcomes [5–11, 27–33], understanding the features of COVID-19 stigma and medical mistrust among PLWH can help elucidate potential impacts of these forces on health-seeking behaviors and guide intervention efforts.

Our findings shed additional light on the prevalence of medical mistrust and conspiracy beliefs related to COVID-19 among PLWH in South Africa. Participants who identified acquiring COVID-19 infection as a major concern during the pandemic demonstrated lower levels of medical mistrust. This association may indicate that individuals with more trust in the healthcare system had a heightened awareness of their risk for acquiring infection, or alternately that those more concerned about infection were more likely to trust public health systems and messaging in order to understand how to avoid exposure to COVID-19. Interestingly, we did not find an association between social media as a source of information on COVID-19 and medical mistrust, counter to previous studies in other settings [36, 45]; however, less than twenty percent of our cohort identified social media as a source of information on COVID-19, highlighting potential differences in dissemination of information regarding the pandemic in South Africa compared to higher-income countries.

We additionally identified characteristics associated with higher COVID-19 stigma, including female gender and higher levels of HIV stigma prior to the COVID-19 pandemic, and found a negative association between television as a source of information on COVID-19 and stigma. An association between gender and health-related stigma has been noted previously, demonstrating more perceived HIV stigma among women than men [56] and differential influences of HIV stigma on care-seeking behavior for men and women [57, 58]. The association of higher HIV stigma at baseline with higher COVID-19 stigma supports the notion that health-related stigma is not an isolated phenomenon specific to a single condition, but rather reflects the intersectionality of multiple health-related stigmas [19, 22] and suggests that structural determinants, such as poverty and systemic racism, may be contributing to multiple health-related stigmas for the same individual or group of people. Interestingly, participants who reported television as a source of information about COVID-19 were less likely to have high COVID-19 stigma; no other source of information was associated with higher or lower stigma or with medical mistrust. The use of television over social media for information in South Africa, with the latter linked frequently to COVID-19 misinformation [40–44] which can in turn fuel stigma, may provide access to more unbiased, comprehensive information on the virus that may counteract stigma.

Overall, endorsement of COVID-19 medical mistrust and conspiracy beliefs was somewhat lower than in recent studies among PLWH and people without HIV in the U.S. and U.K. [47, 55]. Our cohort included only participants who had achieved HIV virologic suppression and clinical stability on ART for at least one year. The baseline characteristics of this cohort (e.g., low rates of competing needs, near-universal self-report of very good or excellent ART adherence) demonstrate that this population is already actively, and successfully, engaging with the healthcare system, and thus potentially less likely to endorse medical mistrust even with regard to a new health condition such as COVID-19. However, a significant proportion of participants, over forty percent, did agree with at least one mistrust item, and one in four participants agreed or strongly agreed that information about coronavirus was being withheld from the public, which was also common among Black Americans living with HIV [47], suggesting the potential impacts of shared histories of intentional disinformation and systemic racism on medical mistrust. Similarly, while a significant proportion of participants disagreed with all items on the stigma scale and thus had the lowest possible summary score for COVID-19 stigma, there was a broad distribution of responses. Notably, over one quarter of participants agreed or strongly agreed that it would be difficult to tell other people if they were infected with COVID-19, and over one out of five people agreed or strongly agreed that they would feel ashamed if they had COVID-19. Both of these items assess internalized stigma, which has been associated with decreased care-seeking for HIV [11] and with negative impacts on mental health in the setting of HIV [50, 59]*.*

Stigma and mistrust have been associated with decreased care-seeking behaviors for other health conditions [5, 8–10, 27–30], for COVID-19 [45, 55, 60], and with COVID-19 vaccine hesitancy [47]. Furthermore, COVID-19 stigma and medical mistrust could negatively impact care-seeking behavior for HIV, if mistrust in the protection against COVID-19 afforded by physical distancing and use of PPE in clinics, mistrust of transmission dynamics of the virus, or stigma associated with healthcare workers or others perceived to be at high risk of COVID-19 infection lead to avoidance of healthcare settings. As many nations worldwide, including South Africa, experience multiple surges of infections and the emergence of SARS-CoV-2 variants, ongoing adherence to mitigation measures to prevent recurrent surges, as well as encouragement of the uptake of effective vaccines, are increasingly important. Interventions to address stigma and medical mistrust related to COVID-19 among vulnerable populations such as PLWH are necessary and will require targeted efforts. Messages disseminated by healthcare providers, opinion leaders, and on television may be particularly effective, as television, a commonly reported source of information on COVID-19, was associated with lower COVID-19 stigma. Acknowledging and addressing structural racism and its impacts on health in this population are critical, including greater investment in healthcare delivery in historically underserved areas, which are often those most affected by HIV and COVID-19, prioritizing underrepresented racial and ethnic groups in healthcare leadership, and ensuring all population groups are represented equally in public health messaging. Additionally, recognizing the intersectionality of health-related stigmas and the differential stigma experienced by women and addressing the history of systematic misinformation on HIV will be important to increasing the trustworthiness of the public health system regarding COVID-19.

## Limitations

This study is one of the first systematic evaluations of medical mistrust and stigma related to COVID-19 in a low- or middle-income country setting, thus there are no existing validated scales or measures in these populations. Our questions, adapted from existing stigma and mistrust scales for HIV and chronic disease or designed specifically for this study, may not comprehensively assess all dimensions of these constructs. Scales to be used more widely to assess COVID-19 mistrust and stigma will require more thorough psychometric evaluations; however, we did not have the capacity to conduct a full assessment in this study. The satisfactory internal consistency of these scales and the consistency of identified associations with other studies suggest, however, that we have measured at least certain dimensions of COVID-19 stigma and mistrust accurately. While many parallels can be drawn between the COVID-19 and HIV epidemics, COVID-19 also differs from HIV in important ways, including a lack of association with sexual risk behavior, possibility of cure, and the rapid accrual and dissemination of information about COVID-19. Our results may not be generalizable to other populations of people living with HIV who are not virally suppressed or clinically stable, those not enrolled in a decentralized ART distribution program, or people without HIV in South Africa. Furthermore, our results have to be interpreted within the circumstances of the early stages of the pandemic and national lockdown in South Africa, as the interviews were conducted over a one-month period spanning only the first two of five stages of the national lockdown. Subsequently, COVID-19 cases in South Africa increased exponentially, global knowledge about transmission dynamics and potential treatments for the infection increased significantly, and due to economic concerns, the South African government has transitioned the nation to lower levels of restrictions. Thus, it is anticipated that levels of stigma and mistrust will also respond in dynamic ways to the changing nature of the pandemic.

## Conclusions

Among people living with HIV enrolled in a decentralized ART delivery program in South Africa, a substantial proportion endorsed at least one aspect of COVID-19 stigma or medical mistrust early in the COVID-19 pandemic. Female gender and higher baseline HIV stigma were associated with higher COVID-19 stigma, while obtaining COVID-19 information from television was associated with lower COVID-19 stigma, highlighting the intersectionality of multiple health-related stigmas and suggesting the potential for mass media to disseminate destigmatizing information about COVID-19. Stigma and medical mistrust could impede efforts to effectively control the COVID-19 pandemic at the population level and targeted interventions are needed.

## Data Availability

Data are not posted online due to potentially identifiable information but are available from the principal investigator upon request.
